# Research Progress and Current Status of Gas–Solid Two-Phase Flow Technology in the Direction of Laser Cladding

**DOI:** 10.3390/mi15101190

**Published:** 2024-09-26

**Authors:** Jianjun Peng, Erhao Zhou, Run Feng, Meng Xue, Junhua Wang, Zhidan Zhong, Xiangchen Ku

**Affiliations:** 1 School of Mechanical and Electrical Engineering, Henan University of Science and Technology, Luoyang 471003, China; zeh@stu.haust.edu.cn (E.Z.); fr@stu.haust.edu.cn (R.F.); xm@stu.haust.edu.cn (M.X.); wangjh@haust.edu.cn (J.W.); zzd@haust.edu.cn (Z.Z.); 2 Collaborative Innovation Center of Henan Province for High-End Bearing, Luoyang 471003, China

**Keywords:** gas–solid two-phase flow, nozzle optimization, powder convergence characteristics, laser cladding

## Abstract

In the process of laser cladding, there are usually problems such as powder plugging and uneven delivery, which affect the quality of the final cladding layer. Therefore, powder convergence characteristics in laser cladding need to be further improved. Gas–solid two-phase flow technology has been widely used in the study of powder flow characteristics because it can precisely regulate the interaction between carrier air and powder flow. In this paper, we systematically review the current status of gas–solid two-phase flow in the field of laser cladding powder, deeply analyze the latest optimization progress of laser cladding nozzle design, and comprehensively explain the key progress of gas–solid two-phase flow technology in improving the uniformity and efficiency of powder field distribution. At the end of this paper, the research results are summarized and a series of prospective prospects are proposed, aiming to provide a valuable reference framework and directional guidance for the subsequent related research.

## 1. Introduction

With the continuous progress of science and technology, laser additive manufacturing technology, with its unique advantages, has overturned the existing pattern of the traditional manufacturing industry and newly stood at the forefront of the manufacturing industry [1,2,3]. As one of the landmark technologies of the new era, laser additive manufacturing has demonstrated a number of characteristics that are difficult to be achieved by traditional manufacturing technologies, including but not limited to its non-contact processing, high energy density, and excellent directionality [4,5,6]. In particular, laser cladding has opened up new frontiers in surface modification research [7,8,9], leading to major technological innovations in key areas such as surface repair and aerospace [10,11,12].

**Figure 1:**
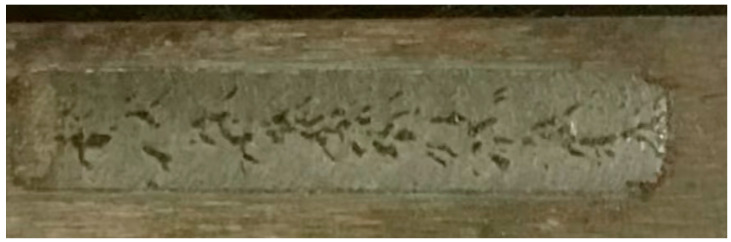
Adverse effects caused by unstable powder feeding.

At present, laser cladding technology is faced with many challenges in powder transportation, including differences in particle properties under different working conditions, differences in powder-blocked pipeline structures, uneven transportation, and low powder utilization [13,14]. These problems not only affect the powder conveying efficiency but also increase the surface roughness of the workpiece [15,16,17], resulting in surface damage of the workpiece [18,19,20,21], as shown in Figure 1. The performance of the workpiece processed by different laser cladding materials will also be different [22,23,24]. The research of gas–solid two-phase flow powder field not only provides an effective solution for the field of additive manufacturing powder but also promotes the innovation of powder transportation technology, which can realize uniform powder coverage on the surface of the substrate, thereby avoiding the problem of local overheating or uneven material deposition and improving the quality of the final product [25,26,27].

Using gas–solid two-phase flow simulation technology, we are able to accurately capture the trajectory of the particles in the pipe and their various characteristics of the distribution during the convergence process in an intuitive visualization way [28,29]. The application of this technology not only provides a scientific basis for the optimization of nozzle design but also significantly reduces the research and development costs. Therefore, it is of great practical significance to explore the study of powder flow characteristics during the laser cladding process to promote the technological progress and application development in this field.

This paper provides an in-depth discussion of the fundamentals of the laser cladding powder field and analyzes the mechanism of gas–solid two-phase flow in the nozzle direction. The article summarizes the recent progress of gas–solid two-phase flow technology in the field of laser cladding, including the current challenges and limitations. Meanwhile, the future development trend is envisioned, aiming to provide guidance and reference for the research in this field.

## 2. Basic Principle of Laser Melting Powder Field and Research Progress of Laser Nozzle

### 2.1. Basic Principles of Laser Cladding Technology

**Figure 2:**
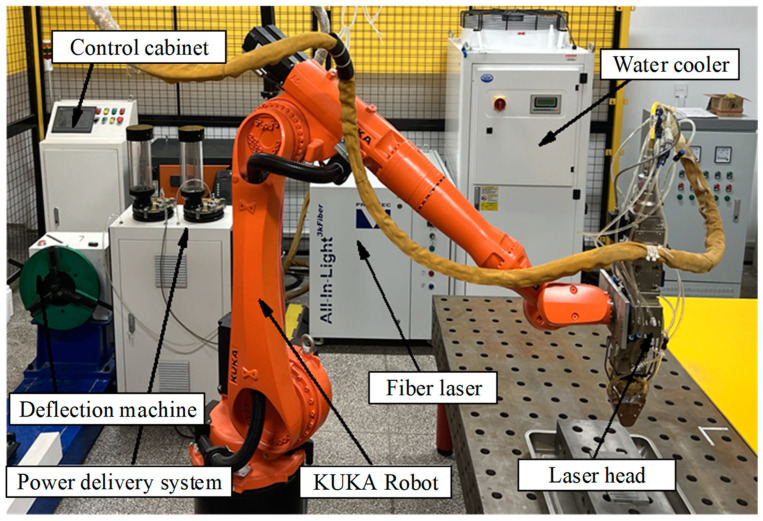
Schematic diagram of the principle of laser cladding process.

Laser cladding technology is an important technology used in additive manufacturing technology for material surface modification technology [30,31,32]. Laser cladding is an important technology for surface modification in additive manufacturing technology [33,34]. Laser cladding technology uses laser as a heat source, prompting the metal powder and the substrate surface to realize instantaneous high-temperature fusion to achieve metallurgical bonding state. Subsequently, through the precise control of the material layer by layer accumulation, to realize the surface repair and enhancement, give the surface of the workpiece more superior performance characteristics [35,36,37]. Figure 2 shows the schematic diagram of the laser cladding process.

**Figure 3:**
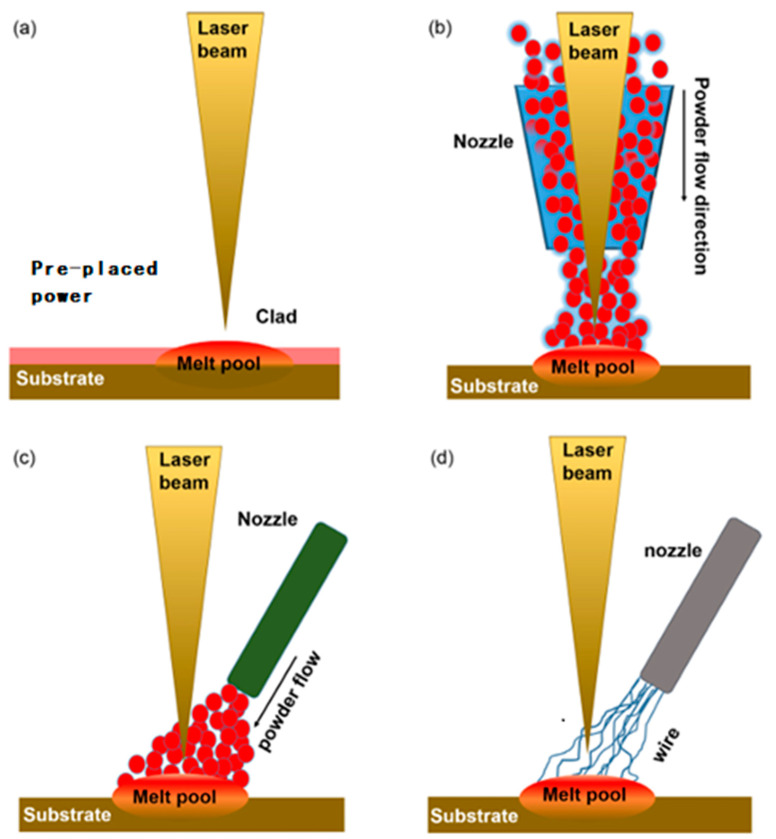
Schematic diagrams of feeding system’s (**a**) pre-placed powder system, (**b**) coaxial powder system, (**c**) off-axis powder system, and (**d**) wire feeding system [39].

The laser cladding process can be categorized into four main types depending on the nozzle structure and powder feeding method, as shown in Figure 3. Figure 3a illustrates a pre-positioned powder system in which the powder is pre-positioned on the substrate and subsequently fused by laser heating. Despite the simplicity of this method, its fusing efficiency is relatively low. Figure 3b shows a coaxial powder system where the powder is delivered along the same axis of the laser beam. Coaxial powder delivery systems are known for their flexibility in parameter adjustment and good directionality and are easy to automate and control and thus have received extensive research attention [38]. Figure 3c shows a side-axis powder system, where the powder interacts with the laser beam by means of lateral powder feeding, but the directionality of this system is somewhat limited. Figure 3d shows the wire feeding system, which uses a metal wire instead of powder; although this method can simplify the operation, sometimes the problem of inaccurate coupling between the laser and the wire occurs [39].

**Figure 4:**
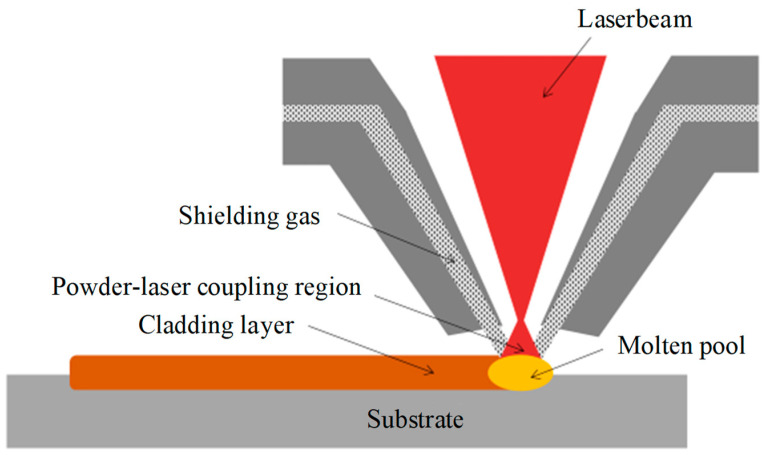
Typical laser cladding process.

Comprehensive comparative analysis of the above four powder feeding methods shows that the coaxial powder feeding method shows significant advantages due to its high degree of flexibility and the ability to accurately regulate the powder feeding speed in any direction, which is conducive to the realization of automated control. A diagram of the coaxial laser cladding process is shown in Figure 4 [40] for visual understanding. This paper systematically reviews the distribution characteristics of the powder field inside and outside the coaxial powder feeding nozzle and discusses the optimization strategy of the nozzle structure and its effectiveness, aiming to provide reference for research and practice in related fields.

### 2.2. Progress in Optimization of Nozzle Structure and Effect on Powder Flowability

As one of the key components in the laser cladding system [40,41,42], the nozzle has a decisive influence on the cladding effect. With the continuous progress of laser cladding technology, many scholars have carried out extensive and in-depth research on powder feeding nozzles and developed a variety of new nozzles, which significantly improve the cladding effect and powder utilization [43]. In this section, the recent progress of nozzle optimization will be outlined, the influence of nozzle design on powder convergence will be discussed, and the performance limitations of the existing coaxial powder feeding nozzles will be analyzed, emphasizing that strengthening the nozzle design is the key in promoting the development of laser cladding technology.

#### 2.2.1. Effect of Different Types of Coaxial Nozzles on Powder Convergence

**Figure 5:**
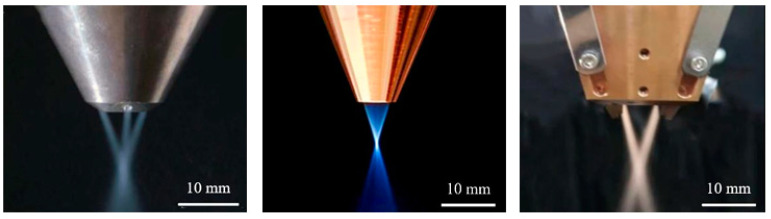
Three types of carrier gas laser melting powder feeding nozzles [45].

Studies by domestic and foreign scholars have shown that different types of powder feeding nozzles have a significant effect on powder convergence [44]. By optimizing the structural design of the nozzle, the airflow characteristics can be improved, which in turn enhances the utilization of powder. As shown in Figure 5, three commonly used coaxial powder feeding nozzle types are demonstrated [45].

Under different working conditions, it is crucial to use powder feeding nozzles with different structures. As shown in Figure 5 (left), the powder feeding effect of multi-way powder feeding nozzles varies according to the design of their pathways. Studies have shown that a three-way powder feed nozzle can aggregate powder more effectively compared to a two-way nozzle [46]. In addition, the increase in the number of powder delivery channels in a multi-way nozzle can help to improve the powder aggregation [47]. Figure 5 (middle) shows an annular powder feed nozzle with a more uniform powder distribution along the circumference at the outlet, thus optimizing the melting process [48,49]. Figure 5 (right) depicts a broadband powder feeding nozzle, which is designed to solve the problem of a small single diameter size and low processing efficiency in conventional cladding technology [50] and effectively improve the cladding efficiency [51].

In different application scenarios, the design types of coaxial powder feeding nozzles show diverse characteristics. Wang W et al. designed a four-hole coaxial powder feeding nozzle with uniform distribution along the axial direction of the center cone hole and investigated the powder flow field characteristics under different outlet shapes and inclination angles by numerical simulation. Experimental validation shows that the design can effectively improve the convergence of powder flow [52]. Liu designed a ring-shaped powder feeding nozzle, and the experimental results show that the ring-shaped laser beam of the nozzle is well coupled with the powder, which can more fully enclose the powder spot and improve the powder utilization [53]. Guo designed a broadband coaxial powder feeding nozzle for high-power laser cladding and analyzed the effects of different feeding angles and outlet gaps on powder convergence through simulation. The experimental validation shows that the powder convergence and utilization are improved under specific nozzle structure parameters [54]. Hu designed a lateral powder feeding nozzle with adjustable powder outlet width and carried out an experimental study on powder utilization, which provided an experimental basis for further optimization of the nozzle [55]. Chen proposed a powder feeding nozzle for broadband laser direct deposition, which adopts a multi-channel outlet structure to control the dispersion angle of the powder and changes the flow field distribution through the internal shunt column to effectively avoid the clogging problem [56].

Different types of powder feed nozzles can produce air–powder streams with specific states, which, when combined with a laser spot of the appropriate shape [57], can significantly improve the convergence properties of the powder and thus enhance the cladding effect.

#### 2.2.2. Effect of Different Nozzle Structural Parameters on Powder Convergence

**Figure 6:**
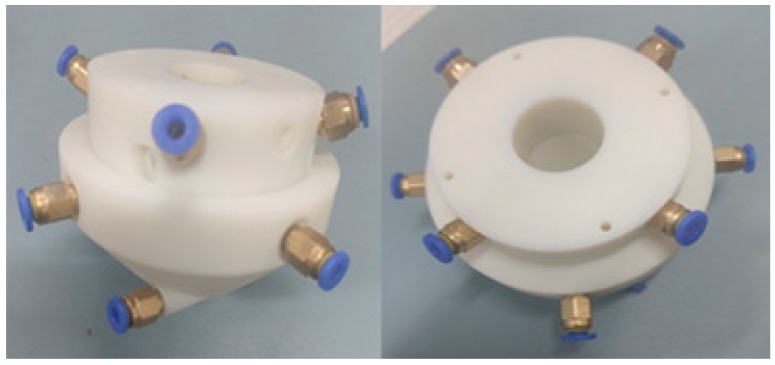
The physical diagram of the optimized laser cladding coaxial powder feeding nozzle [61].

Nozzles are designed to limit particle movement through the interaction of the powder with the pipe wall, thus reducing the degree of dispersion of the powder as it is ejected. In order to avoid disordered splashing of powder particles at the nozzle outlet, the nozzle structure needs to be carefully constructed, especially to form a proper taper design to ensure that the powder particles have excellent convergence characteristics after being ejected from the nozzle. At present, many scholars focus on the optimization of key structural parameters of the nozzle to enhance its powder feeding performance. In order to investigate the influence of the nozzle structural parameters on the concentration of powder convergence and the size of the powder spot, Zhao uses analog simulation software to conduct orthogonal simulation analysis of key structural factors, such as powder channel inclination, outlet clearance, vertical height, and shrinkage angle of coaxial powder feeding nozzle. By integrating data of powder concentration and powder spot size, the optimal combination of structural parameters is determined. The simulation results of powder concentration convergence and powder spot size are in good agreement through experiments [58]. Wang, for the four-way powder feeding nozzle, investigated the powder flow characteristics under different powder feeding port sizes, powder feeding angles, and contraction angles through numerical simulation and pointed out that the convergence performance of the powder flow can be significantly improved under specific structural parameter configurations [59]. Ju used the response surface method for composite design, analyzed the relationship between the main structural parameters of the nozzle and the characteristics of the air–powder flow in depth, and constructed the corresponding mathematical model to perform the regression fitting. Subsequently, the optimized nozzle was applied to laser cladding experiments, and the results showed that the error between experimental data and simulation predictions was less than 9%, which verified the effectiveness of the optimization method [60]. Aiming at the problems of low powder utilization and easy clogging of the existing nozzles, Su innovatively combined the response surface method with the BP neural network optimized by genetic algorithm to comprehensively optimize the structural parameter combinations of the ring nozzles. The optimized ring-shaped laser cladding coaxial nozzle structure is realized by photosensitive resin material printing technology, and the experimental results show that the predicted values of the optimized parameter combinations are in high agreement with the measured values, which significantly improves the powder feeding effect. The optimized nozzle is shown in Figure 6 [61].

The above scholars are committed to improving the effect of powder through the numerical simulation method for the convergence, optimization of the nozzle structural parameters, and different structures of the nozzle in the optimization of the best parameter combinations. In view of the diversity of actual working conditions, in order to effectively reduce costs, subsequent research should continue to rely on simulation technology for different powder feeding nozzles to fine-tune the structural parameters in order to determine the optimal structural parameters of the respective working conditions and then guide the design and manufacture of nozzles.

#### 2.2.3. Research Progress in Laser Nozzle Optimization

Since the structure of the powder feeding nozzle directly affects the convergence characteristics of the powder, which in turn affects the quality of laser cladding, a series of scholars have widely carried out research on the optimization of the structure of the coaxial powder feeding nozzle for laser cladding and have deeply explored the multi-dimensional influence of the nozzle structure parameters on the convergence of the powder.

Gao took the lead in designing the structure of the coaxial powder feeding nozzle and analyzed the movement of powder inside the nozzle [62]. Zhang, on the other hand, innovatively developed a coaxial powder feeding nozzle integrating guided airflow, water cooling, and other functions through structural optimization, and experimental validation showed that the nozzle can be directly applied in the manufacturing process of metal parts [63]. Zhang et al. proposed an innovative solution to address the shortcomings of the existing nozzles, i.e., the design of four double-layer powder feeding tubes distributed uniformly and symmetrically around the nozzle, realizing the function of four-way coaxial powder feeding [64]. Zhou designed two types of coaxial powder feeding nozzles, namely, hole type and ring type, and successfully built a prototype and verified the reliability of these two types of nozzles through a series of experiments [65]. Li used CFD technology to conduct detailed numerical simulations of the powder feeding flow field, explored the characteristics of the gas–solid two-phase flow field, powder trajectory, focusing concentration, and radius under various conditions, and accordingly designed a laser coaxial powder feeding nozzle with an adjustable focal point and vertically removable [66]. Yang, focusing on simplicity, designed and fabricated a no-assembly coaxial powder feeding nozzle for laser cladding [67]. Based on the theory of gas–solid two-phase flow, Zhu numerically simulated a wide-band lateral powder feeding nozzle, successfully improved the nozzle structure with the uniformity of powder concentration distribution as the optimization objective, and manufactured a width-adjustable powder feeding nozzle, which was experimentally verified by the validity of his model [68]. Duan designed a nozzle with a contraction-shaped flow channel and adopted a double-side powder feeding method, which effectively solved the problem of irreversible powder movement direction in single-side powder feeding [69]. Zhenjiang Zhang established a simulation model of the flow field of a three-dimensional powder feeding nozzle and applied the response surface method to optimize the structural parameters of the coaxial powder feeding nozzle for laser cladding. The experimental results show that the optimized nozzle exhibits better performance in both the powder feeding test and the single-pass laser cladding test, and the powder convergence is significantly improved [70]. Finally, Yi focused on the research of adjustable coaxial powder feeding nozzle, revealed the powder distribution law, and designed a coaxial powder feeding nozzle that is suitable for both air and gravity feeding and is vertically removable [71].

**Figure 7:**
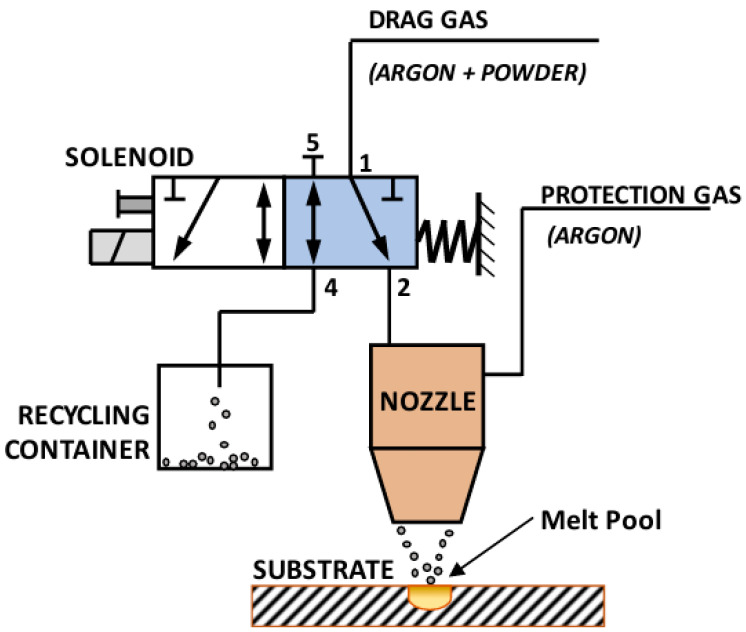
Scheme of the powder flux regulation system where the solenoid is in position 1 [74,75,76,77].

Tabernero presented an innovative powder flow model that accurately predicts powder distribution patterns, particle velocities, and their trajectories with high agreement between experimental measurements and model predictions. This achievement provides a powerful tool for evaluating the optimal parameters and designing the coaxial nozzle geometry to optimize the particle flux [72]. Oliveira’s study further pointed out that the velocity of the powder particles needs to be maintained in a specific interval to avoid excessive laser power to melt the powder particles due to the high speed, which may affect the stability of the melt pool. Therefore, an in-depth analysis of the temperature field during the laser cladding process is particularly important [73]. Arrizubieta et al. successfully developed a smart nozzle by integrating various sensing and control systems into the nozzle. The nozzle not only automatically adjusts the laser power based on real-time measurements of the melt pool temperature but also relies on advanced algorithms to accurately regulate the powder flow rate to the processing area, ensuring that the powder flow rate per unit of surface area deposited remains constant, which significantly improves the stability and efficiency of the process [74,75,76,77]. The specific realization of the powder flow regulation system is shown in Figure 7.

**Figure 8:**
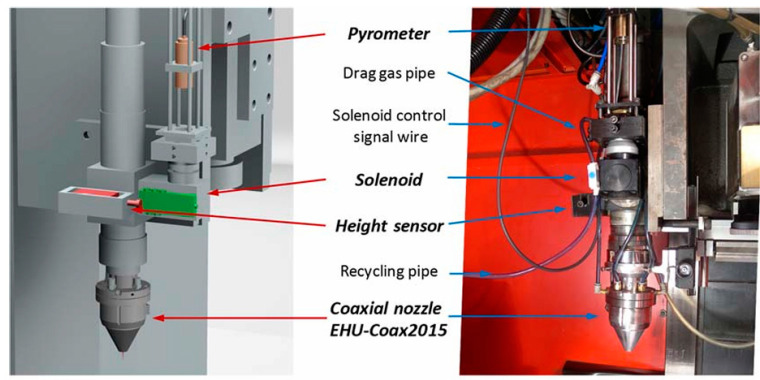
Scheme and photo of the EHU-Coax2015 nozzle with the developed sensing and control systems [74,75,76,77].

The smart nozzle is shown in Figure 8.

Cassiano Bonin et al. innovatively applied an intelligent neural sensor combined with a machine learning model aimed at accurately predicting the actual variation in powder flow rate and particle trajectory clogging phenomena during laser cladding. This study not only deepens the understanding of powder clogging events during laser cladding but also highlights the great potential of machine vision technology in laser applications. In addition, they propose a specific strategy for deploying smart sensors in production environments, providing a novel and effective solution to a common problem in this field [78].

**Figure 9:**
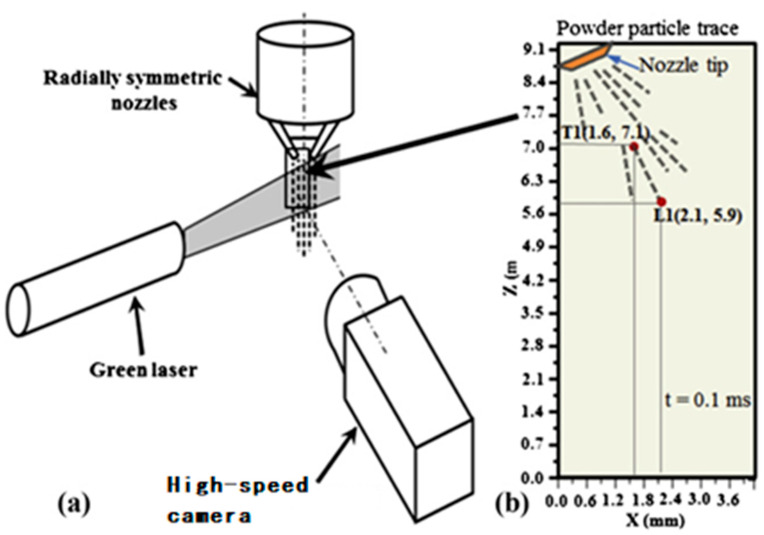
Schematic of velocity measurement (**a**) and velocity measurement scheme (**b**) [86].

The powder field in the laser cladding process is very complex, and the structure and size of the nozzle directly determine the trajectory and spatial distribution characteristics of the powder particles [79]. Due to the difficulty of direct observation of the powder particle movement inside the pipe, three-dimensional flow simulation of the nozzle becomes a key link to optimize the design of the nozzle structure. Liu showed that the traditional nozzle convergence effect is poor, easy to clog, and other problems exist in structural design and optimization, and through simulation, they performed an in-depth analysis of the powder particles in the flow field of the force state and trajectory. Experimental verification shows that the optimized nozzle significantly improves the circumferential dispersion uniformity of powder thus enhancing the powder feeding effect [80]. Zhu, on the other hand, focused on the design of coaxial powder feeding nozzles and revealed the significant influence of the nozzle geometry on the intensity distribution of the cladding layer through simulation [81]. Tian developed a new type of laser coaxial powder feeding nozzle, which adopts the carrier gas unloading and then powder feeding technology. The influence of different structural parameters on the concentration distribution and convergence performance of the powder flow field was systematically investigated through simulation, and the experimental results show that the nozzle significantly improves the convergence of the powder flow and effectively enhances the utilization of the powder [82,83]. Lin conducted numerical simulations of the powder flow using Fluent software for the nozzles with different outlet arrangements and found that a specific arrangement can significantly improve the concentration of the powder concentration [84]. Liu constructed a gas–powder flow model through three-dimensional computational fluid dynamics and deeply explored the influence of the nozzle outlet geometry and other factors on the powder flow characteristics [85]. In addition, Prabu Balu et al. also established a CFD-based powder flow model specifically for characterizing the flow behavior of Ni-WC coaxial powder. They investigated in detail the effects of nozzle tilt angle, outlet diameter, and elastic recovery coefficient on the powder flow concentration distribution and innovatively used three imaging techniques to capture the powder flow reality, which provided rich data support for understanding the powder flow mechanism. A velocity measurement system is shown in Figure 9 [86].

Grigoryants and his team constructed a mathematical model of powder flow in a coaxial nozzle, determined the optimal geometric parameters of the nozzle based on CFD technology, and discussed in depth the influence of these parameters on the powder flow characteristics [87]. Karim proposed a numerical model to accurately simulate the flow state of powder flow released from a coaxial nozzle using Fluent CFD software [88]. Heng Pan used a stochastic particle-wall collision model for detailed simulation of gravity-driven powder flow to further enrich the theoretical study and used a random particle-wall collision model to conduct a detailed simulation of gravity-driven powder flow, which further enriches the theoretical study of powder flow behavior [89,90]. Zhang et al. simulated the effects of powder chamber inclination angle and three protective gases on the powder aggregation performance by Fluent software, and combined with an orthogonal experimental method, they revealed that the external protective gas has the most significant effect on the aggregation of powders. Based on this finding, they optimized the nozzle structure and successfully verified the optimization effect experimentally [91]. Smurov’s numerical simulation work revealed that the trajectory, temperature, and mean mass flow of powder particles inside the nozzle were not only governed by the nozzle geometry but also closely related to the type of collision between the particles and the nozzle wall [92]. Pan focused on the gravitationally driven powder feeding mode, investigated the powder flow characteristics, focused on the gravity-driven powder feeding mode, investigated the combined effects of the powder flow characteristics, nozzle shape, and protective gas settings on the powder flow field, and constructed a three-dimensional nozzle numerical model to predict the trend of the powder flow concentration [93]. In addition, Bi innovatively developed a compact laser cladding head with integrated sensors and coaxial powder feeding function, which provides new ideas for the intelligent and integrated development of laser cladding technology [94].

The above nozzle optimization studies are based on the Ideal assumption of continuous and uniform powder supply In the simulation; however, in the actual powder feeding process, the phenomenon of powder plugging occurs from time to time, which leads to uneven powder feeding. As the core component of the laser cladding system, many scholars designed and optimized a variety of new nozzles, which significantly improved many problems in the powder feeding process. Despite these remarkable results, the powder utilization rate still needs to be further improved. In view of the specific needs of different working conditions for the design of powder feeding nozzles, their design and optimization are still the focus of the current research on laser cladding technology. Therefore, in-depth exploration of the performance optimization mechanism of laser nozzles is of great significance in promoting the development of laser cladding technology.

## 3. Numerical Simulation of Gas–Solid Two-Phase Flow

In laser cladding technology, the powder convergence characteristics are directly related to the powder utilization, which is one of the key factors affecting the quality of the cladding layer [95,96,97]. Although some researchers tried to optimize the process parameters to improve the quality of the cladding layer, this method is often accompanied by high experimental cost and time consumption [98]. In contrast, the numerical simulation of gas–solid two-phase flow shows unique advantages, which can accurately simulate the flow field information under the given boundary conditions and provide strong support for the analysis of powder convergence properties. By combining numerical simulation with experimental validation, the depth and persuasiveness of the study can not only be significantly improved, but also the cost of the test can be effectively reduced and the research process can be accelerated [99,100]. Therefore, the numerical simulation of gas–solid two-phase flow has an inestimable value in the study of laser cladding powder transport characteristics.

### 3.1. Basic Principles of Gas–Solid Two-Phase Flow and Current Research Status

#### 3.1.1. Gas–Solid Two-Phase Flow Theory

The principle of gas–solid two-phase flow is that the particles are driven by a gas [101,102]. The principle of gas–solid flow is that the particles flow under the impetus of a gas [103,104]. However, in the complex process of laser cladding, it is not sufficient to consider only the push of the gas on the particles to fully characterize their flow. In fact, the intrinsic properties of the powder material, such as density, shape, surface roughness, and dryness/humidity, have a significant effect on the flow behavior. In addition, the mutual collision between particles, the friction between particles and the inner wall of the pipe, and the geometrical properties of the nozzle structure, such as diameter, length, curvature, etc., are also important factors determining the powder flow characteristics. Therefore, in the study of gas–solid two-phase flow in laser cladding, it is necessary to comprehensively consider the above factors.

#### 3.1.2. Interphase Coupling of Gas–Solid Phases

Currently, there are three main mainstream methods in the field of numerical simulation of gas–solid two-phase flow: the Eulerian two-fluid model, the Eulerian–Lagrangian model, and the coupled CFD-DEM model that is receiving increasing attention.

**Figure 10:**
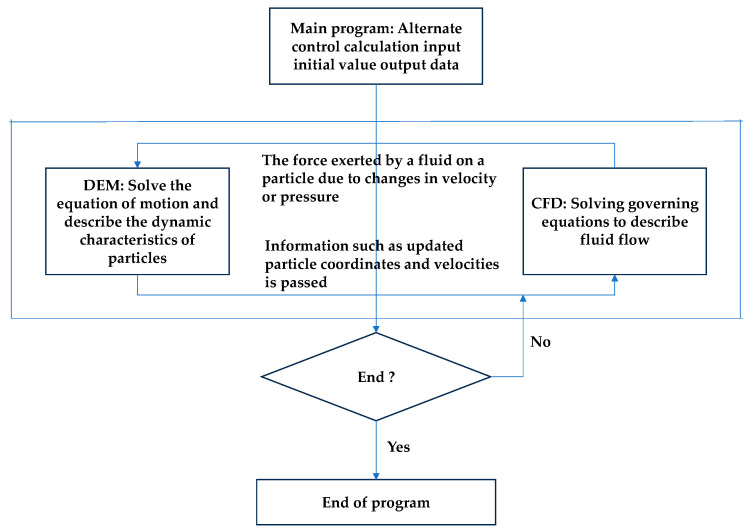
CFD-DEM coupling flow chart [111].

1. Eulerian two-fluid model: This model treats both gas–solid phases as continuous media and assumes that they fill the entire flow field. By applying the continuity and momentum equations of the continuous medium to the two phases, the computational complexity and computational time are significantly reduced and the computational efficiency is improved [105].
2. Eulerian–Lagrangian model: In this model, the powder feed gas is considered as a continuous phase, while the powder particles are treated as a discrete phase. Given that the particle phase does not have fluid properties and the volume fraction is small, the model ignores the interaction and influence of the particles relative to the continuous phase [106]. The model is capable of accurately tracking the trajectory of each particle, providing a powerful tool for analyzing particle dynamics.
3. CFD-DEM coupled model: This model combines the advantages of computational fluid dynamics (CFD) and Discrete Element Method (DEM), in which CFD is used to solve the gas–phase flow, while DEM focuses on the dynamic simulation of the particle phase [107,108]. This model cannot only visualize the flow state, shape, and size of the particles and other characteristics but also accurately simulate the collisions between particles trajectories and other complex behaviors [109,110]. Figure 10 shows the flow chart of the coupled CFD-DEM model, which further clarifies its workflow and advantages [111].

**Figure 11:**
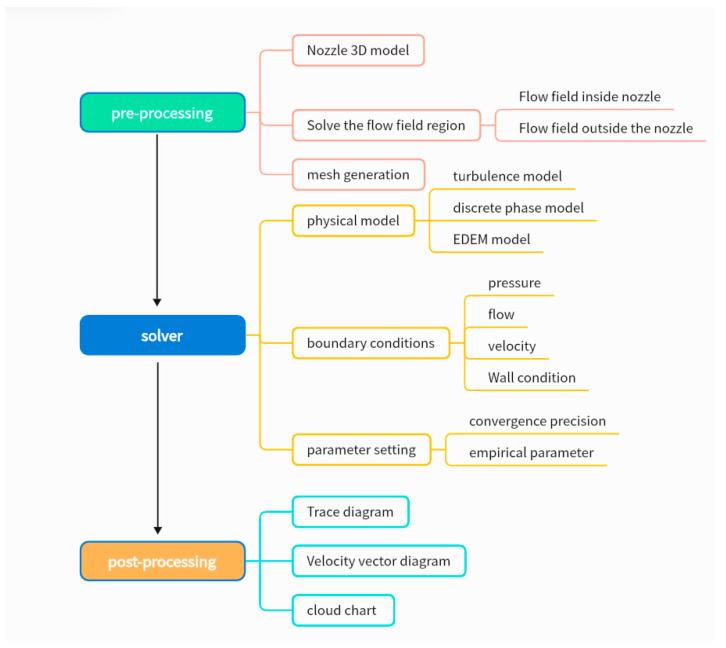
FLUENT flow chart.

Gas–solid two-phase flow simulations are usually simulated with FLUENT, the flow chart of which is shown in Figure 11.

In summary, choosing a suitable solution model can not only make the calculation of the powder field more accurate but also save the simulation calculation time and improve the work efficiency.

#### 3.1.3. Current Research on the Convergence of Air–Powder Streams in Laser Cladding Nozzles

The laser cladding powder field can be divided into two stages, i.e., the flow field inside the nozzle and the flow field outside the nozzle. Since the flow field inside the nozzle is not visualized and the powder distribution characteristics are difficult to understand from experiments, it needs to be further studied with the help of simulation software. At present, domestic and foreign scholars have confirmed the high accuracy and reliability of the gas–solid two-phase flow theory in the analysis of powder transport flow field through extensive research and experimental validation, providing a solid theoretical foundation for the research in related fields [112].

**Figure 12:**
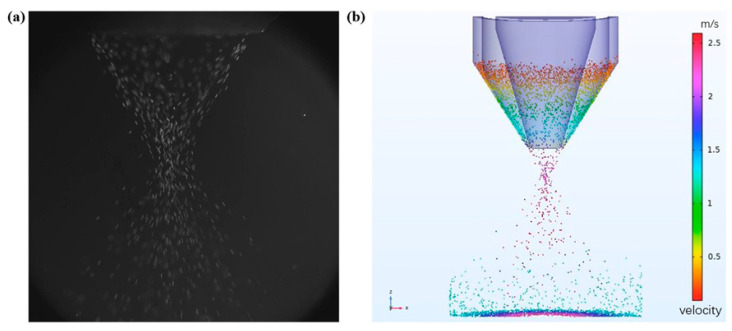
Powder flow characteristics in laser cladding. (**a**) Captured by high-speed camera; (**b**) simulation results [116].

Rui feng Yan established a three-dimensional numerical model of powder flow, analyzed the two-phase flow by Eulerian method, calculated the pressure of powder flow and powder concentration distribution, and explored the law of optical powder coupling [113]. The effect of nozzle structure parameters on powder flow was investigated. Jin established a simplified physical model of powder flow and numerically analyzed the coaxial powder flow using Eulerian two-fluid method [114]. The coaxial powder flow was analyzed numerically by Eulerian two-fluid method. Xiaoxin Jin constructed a three-dimensional model of powder, analyzed the powder field by Eulerian–Lagrangian method and convergence characteristics, and found that the powder utilization rate increases with the increase in air volume and particle velocity and the powder with a smaller particle size has a higher powder utilization rate [115]. Liu et al. established a three-dimensional finite element model describing the multi-physics field of coaxial powder feeding laser cladding, and the powder flow characteristics in laser cladding are shown in Figure 12 [116].

**Figure 13:**
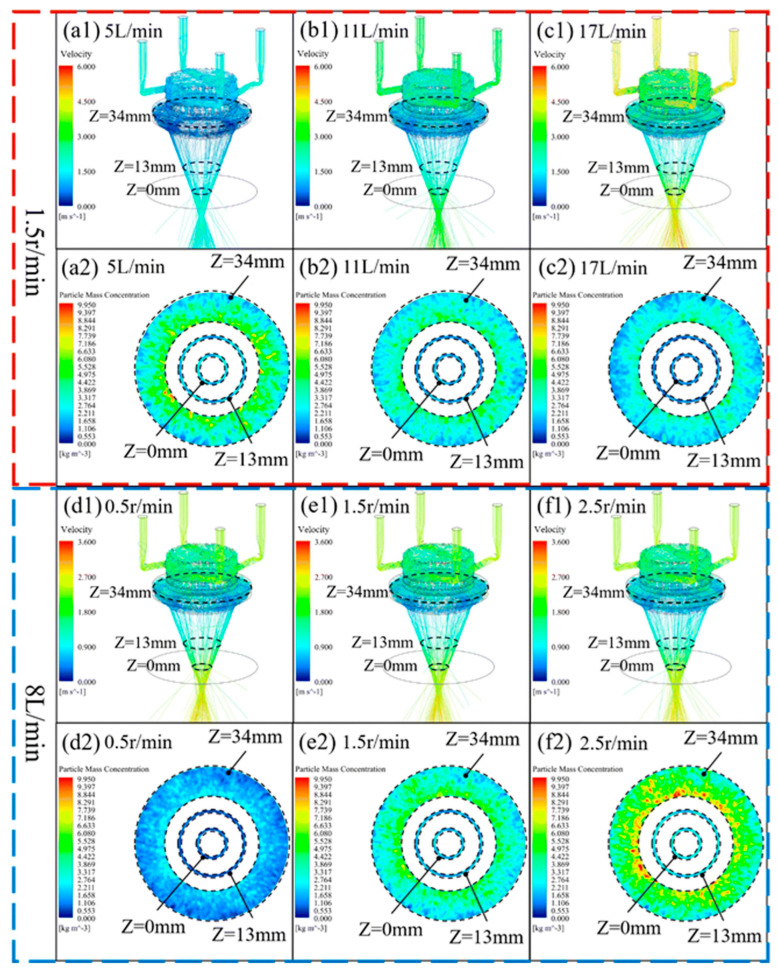
(**a1**–**f1**) The three-dimensional morphology of the powder jet inside the nozzle, and (**a2**–**f2**) the powder concentration distribution of different cross-sections corresponding to (**a1**–**f1**), respectively [118].

He investigated the effect of the radius of curvature of the edge of a solid part on the amount of powder injected into the molten pool at the edge position by the Euler–Lagrange method [117]. Xia developed a new type of nozzle with a four-channel powder feeding transformed into an annular distribution inside the nozzle, established a three-dimensional hydrodynamic model, and described the kinetic behavior of Fe-based powder with a discrete phase model (DPM). The three-dimensional morphology of the powder jet inside the nozzle is shown in Figure 13 [118].

In order to study the effect of different process parameters on the convergence of the powder flow field, Guo used a discrete phase model to simulate it. It is found that the powder convergence characteristics are affected by the carrier gas flow rate and the powder feeding volume. The powder concentration at the focus of powder convergence is negatively correlated with the carrier gas flow rate and positively correlated with the powder feeding volume [119].

The above gas–solid two-phase flow analysis and calculation models mainly include the Eulerian two-fluid model and the discrete phase model, which can roughly reveal the trend of the powder field but are limited by the complex collision behavior between the powders and the characteristics of the powders [120], which makes it difficult to accurately portray the actual distribution of the powder flow in three-dimensional space. In order to compensate for this deficiency, some researchers turned to CFD-DEM coupling technology to realize the accurate simulation of the powder particle collision process. Specifically, Chunyu Li used the EDEM simulation platform to systematically investigate the influence of different powder feeding hole angles on powder convergence characteristics, which provides a scientific basis for optimizing the design of powder feeding holes [121]. Fan, on the other hand, comprehensively analyzed the combined effects of powder feeding process parameters and powder intrinsic parameters on powder convergence by constructing a CFD-DEM coupled nozzle simulation model and introducing the response surface method [122]. In addition, based on CFD-DEM technology, Fan successfully constructed a three-dimensional coupled model of the laser cladding process and deeply investigated the influence mechanism of different process parameters on powder utilization. Through the implementation of orthogonal ANOVA, the powder feeding volume was pointed out as a key factor affecting the powder utilization rate, and the prediction accuracy and reliability of the model were further verified by experiments [123]. These studies not only enrich the theoretical basis of laser cladding technology but also provide strong support for process optimization in actual production.

At present, in the research field of gas–solid two-phase flow in powder field, although the Eulerian two-fluid model and the discrete phase model dominate, few studies have used the CFD-DEM coupled model for in-depth analysis. In view of the significant advantage of the CFD-DEM model in portraying the particle motion characteristics, its ability to accurately describe the powder field is more prominent. Therefore, the CFD-DEM coupled model is expected to be a more suitable and powerful analytical tool for the future study of gas–solid two-phase flow powder field.

### 3.2. Powder Flow Characterization

In the laser cladding process, due to the existence of energy momentum and mass transport physical processes in the powder flow, which directly determine the size, accuracy, and performance of the manufactured parts, an in-depth study of its powder flow characteristics is required [124,125]. The powder flow characteristics need to be studied in depth.

#### 3.2.1. Effect of Particle Collisions on Powder Flow Properties

Due to the powder feeding process, the powder particle group in the nozzle wall will cause contact collision and generate force. The collision between the powder particles and the nozzle wall is a non-complete elastic collision, and there is friction. Under certain circumstances, such as charged particles or particles not being completely dry, there is also a non-contact force between the particles [126]. In some cases, such as charged particles or incompletely dried particles, there are also non-contact forces between particles. Currently, the most widely used methods for calculating particle collision forces in laser cladding technology are the hard-sphere model and the soft-sphere model, and a comparison of the characteristics of the two is shown in Table 1.

**Figure 14:**
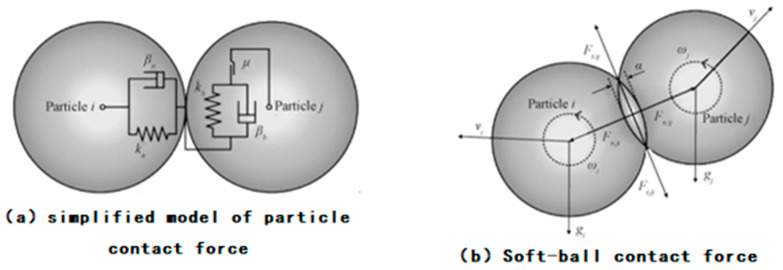
Soft-sphere model inter-particle contact force model [130].

The hard-sphere model is based on the assumption that the collision is instantaneous and focuses only on the change in velocity after the collision, ignoring microscopic details such as particle deformation [127]. In contrast, the soft-sphere model takes into account the elasticity of the particles, estimates the force on each particle by calculating the deformation, damping, and sliding effects of the particles, and updates the velocity of the particles with a time-integrated model [128,129]. The computational complexity of the soft-sphere model is relatively high due to the fact that it requires real-time monitoring and evaluation of potential collisions between particles in a specified area. Figure 14 visualizes the computational model of the contact forces between particles in the soft-sphere model [130].

**Figure 15:**
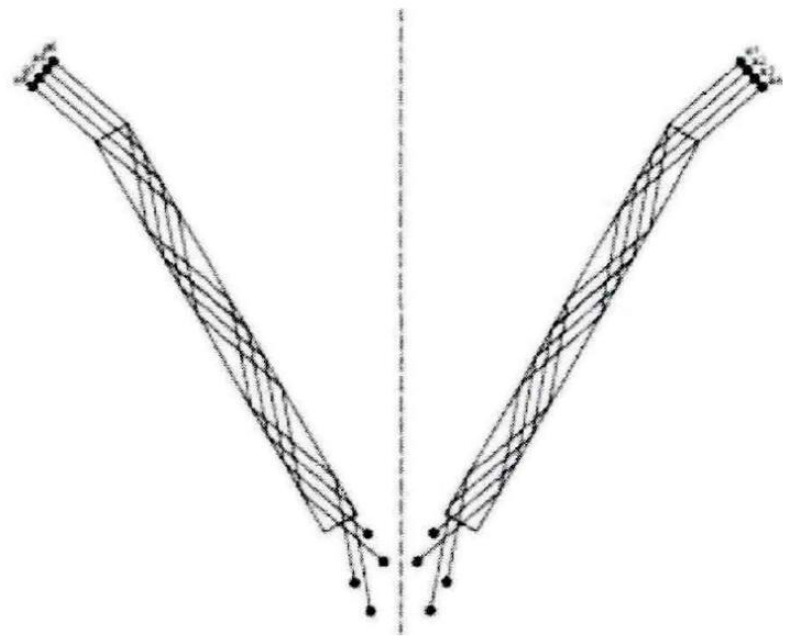
Trajectory of particle movement in powder cavity [131].

One of the key parameters of the hard-sphere model is the elastic recovery coefficient, which is defined as the ratio of the velocity of the particles after collision with the pipe wall to the velocity before collision and is limited to a range of values between zero and one. Theoretically, when the elastic recovery coefficient is equal to one, it means that there is no energy loss during the collision; however, this does not hold true in practice. On the contrary, the closer the value of the elastic recovery coefficient is to one, the smaller the energy loss after the collision. In order to investigate the trajectory of powder in the powder cavity, Huang Dong established a model of powder collision in the powder cavity under certain assumptions, as shown in Figure 15 [131].

**Figure 16:**
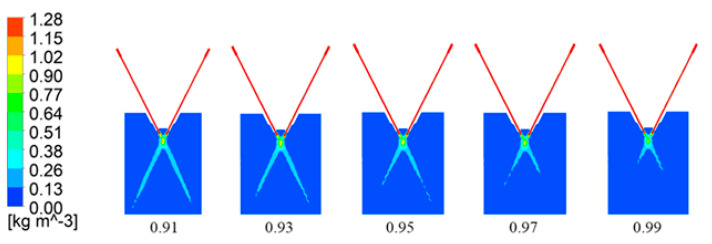
Cloud view of powder concentration distribution in vertical direction [132].

Powder particles in the powder cavity and the pipe wall are constantly colliding, resulting in a loss of energy and momentum, and the trajectory is constantly changing. This results in the final outflow angle of the powder particles entering the nozzle being different from the angle of incidence, and the powder disperses when it is ejected from the nozzle outlet. Yin studied that the elastic recovery coefficient is between 0.91 and 0.99 and when the elastic recovery coefficient is smaller, the convergence is better. Figure 16 shows the powder flow concentration distribution in the vertical direction of the nozzle with different elastic recovery coefficients [132].

Yichen Huang’s study focused on the elastic recovery coefficient in the range of 0.91 to 0.99 and found that a decrease in this coefficient leads to a corresponding reduction in the size of the powder patch [133]. Guo further explored the effect of the elastic recovery coefficient on the flow field outside the four-way nozzle in this range, pointed out that under low elastic recovery coefficient, the powder particles lose significant energy after bouncing several times on the inner wall of the pipeline, and slowed down the velocity at the exit, which reduces the dispersion angle and facilitates the convergence of the powder flow [134]. Feng proposed an equivalent algorithm to simplify the powder collision process inside the nozzle and effectively predicted the overall morphology and spatial density distribution of the powder bundle [135]. By establishing a three-dimensional powder flow model, Qi Zhang reveals that a decrease in the elastic recovery coefficient enhances the concentration of the powder flow at the focal point, reduces the dispersion phenomenon, and improves the convergence performance of the gas–powder flow [136]. Polyanskiy’s validation work shows that the velocity change in the particles is limited after they leave the pipe and that the dominant factor of the velocity change is the collision of powder particles with the inner wall of the powder chamber [137]. The study of Duw points out that when the elastic recovery coefficient is lower than 0.90, the simulation results have a large deviation from the actual situation, and the recommended value range is usually from 0.91 to 0.99 [138]. Kovaleva constructed a three-dimensional air–powder transport model for nozzles with different geometries in coaxial laser cladding and analyzed in depth the transport characteristics of the powder and the wall of the nozzle under different collision conditions [139]. Liu et al. established a three-dimensional numerical model for the given coaxial nozzle, which takes into account the collision between the nozzle and powder, and used it to calculate the particle velocity of the powder particles and the wall of the chamber. He developed a three-dimensional numerical model for a given coaxial nozzle, considering the collision between the nozzle and the powder, for calculating the particle trajectory and the powder concentration distribution, and explored the effects of particle size and recovery coefficient on the powder mobility [140]. Hao Liu and his team developed a numerical model of gas–powder transport for coaxial powder feeding laser cladding in order to improve the convergence of powder beams and systematically analyzed the effects of different powder parameters and rebound coefficients on the convergence of the powder flow [141,142]. Zhang et al. developed a three-dimensional numerical model of a coaxial powder feeding laser cladding nozzle, which comprehensively simulated the gas dynamics and powder transport characteristics inside the nozzle, including the collision behavior of the powder, the trajectory, the velocity distribution, the powder flow outside the nozzle, as well as the structure and convergence characteristics of the powder flow outside the nozzle [143].

Due to the narrow nozzle duct, the powder convergence effect and inter-particle collision behavior are highly constrained by multiple factors such as powder material properties, particle size, and shape. Simply relying on the elastic recovery coefficient to quantify the velocity change before and after powder collision in the nozzle is difficult to fully incorporate these complex factors, resulting in a gap between the simulation results of powder convergence and the actual situation [144]. At present, although the above studies have contributed a lot, few scholars have explored the soft-sphere model in depth. Compared with the hard-sphere model, the soft-sphere model shows higher accuracy in portraying the collision characteristics of particles, which can more realistically reflect the motion state of particles in the dynamic process. This feature is particularly important for the in-depth analysis of powder convergence characteristics and also provides a solid theoretical basis and a powerful analytical tool for improving the utilization of powder.

At present, the gas–solid two-phase flow powder field is devoted to studying the collision characteristics of particles, and little is known about the flow of the molten pool after the action of powder and laser and the situation of the powder entering the molten pool. The gas–powder optical three-phase flow can better understand the flow of the powder flow and the flow of the molten pool and obtain the three-dimensional shape of the molten pool, so as to understand the entire process of laser cladding.

#### 3.2.2. Effect of Process Parameters on Powder Flowability

In terms of powder feeding process parameters, a series of scholars have also conducted a series of studies on process parameters such as carrier gas flow and powder feeding volume [145].

**Figure 17:**
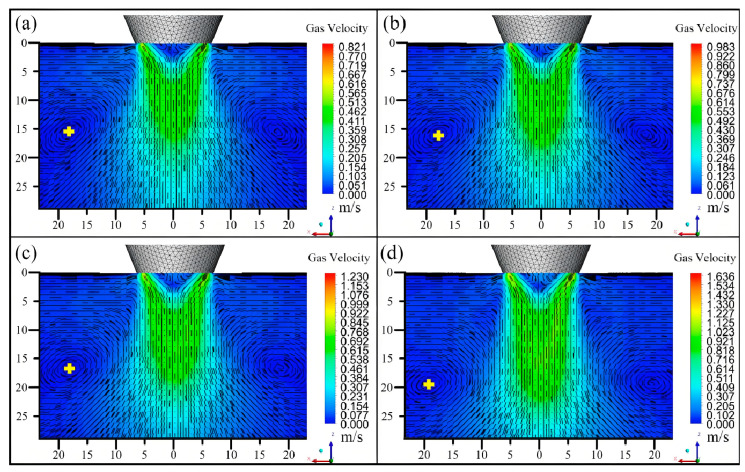
Velocity streamlines for different carrier gas flow rates at 4.0 g/min: (**a**) 2.0 L/min; (**b**) 2.4 L/min; (**c**) 3.0 L/min; and (**d**) 4.0 L/min [153].

Lu Wang analyzed the effect of carrier gas flow on powder temperature, velocity, and laser attenuation [146]. Tuo Shi established a three-dimensional numerical model of a new type of composite nozzle for intra-laser powder and wire feeding and investigated the effect of carrier gas flow rate and other parameters on the powder flow field [147]. Chu analyzed the effects of different process parameters on powder concentration and morphology and found that increasing the carrier gas flow rate is beneficial to the aggregation of powder particles. At the same time, with the increase in the powder feeding voltage, the melting area will increase, and the utilization rate of powder will also be improved [148]. Wang, in the process of laser cladding, in the interaction between the laser beam and the powder stream showed the laser energy and the powder mass transport as having an important effect on the molding accuracy and mechanical properties [149]. Liu used the numerical model of gas–solid two-phase flow theory to simulate the powder flow in the coaxial laser cladding process and investigated the relationship between the gas flow rate and the powder concentration distribution in order to optimize the powder feeding process [150]. Based on the gas–solid two-phase flow theory, Wang et al. established a numerical model of coaxial powder feeding by CFD and investigated the powder flow characteristics and the trajectory of powder particles in the flow field outside the nozzle. The optimization of the powder feeding volume and the carrier gas velocity was carried out, and the results showed that the powder utilization rate was better improved and provided new ideas for the structural design of the nozzle. The results were further verified by experiments, which made the results more convincing [151]. Deng used the numerical simulation of gas–solid two-phase flow and found that with the increase in the carrier gas flow, the velocity of the particles gradually increased, the concentration of focusing gradually decreased, and smaller particles were more likely to cause the dispersion of the particles [152]. Yang Lin designed a new double-ring coaxial nozzle, through the gas–solid two-phase flow numerical simulation of the powder transport characteristics of the study, and found that with the increase in the carrier gas flow rate, the rate of powder particles in the nozzle increased, and the convergence of the stronger, different carrier gas flow rate of the velocity streamline cloud is shown in Figure 17 [153].

Different process parameters have different effects on the convergence characteristics of the powder flow [154,155], for example, the velocity of the carrier gas flow rate is too large or too small, which is not conducive to the convergence of the powder flow, so it is necessary to take the range of values of the process parameters in numerical simulation and then carry out the experimental validation in order to ensure the experimental process has high efficiency and maximize the use of resources to avoid unnecessary waste.

## 4. Conclusions and Outlooks

This paper provides a comprehensive review of the application of gas–solid two-phase flow in laser cladding powder fields, focusing on the optimized design of nozzles and gas–solid two-phase flow characteristics as two dimensions, and provides an in-depth discussion of the research progress in this field. Numerical simulation of gas–solid two-phase flow, as an intuitive and economical method, provides a strong support for understanding the intrinsic mechanism of the powder flow process in laser nozzles. However, it is undeniable that the description and solution of particle collisions as well as the optimization and design of nozzles are still difficult to understand. For the outlook of future works on powder fields, the following directions can be emphasized.

(1) Deepen the study of particle collision mechanism: Through the introduction of CFD-DEM coupled model, we systematically investigate the effects of different particle size distributions, non-complete spherical particle morphologies, and the complex collision mechanisms between particles on the convergence characteristics of powders. This will help to simulate the actual working conditions more accurately and improve the accuracy and reliability of numerical simulation.

(2) Intelligent design of powder feeding nozzles: Using numerical simulation technology and real-time sensing technology to promote the intelligent design and development of powder feed nozzles, real-time monitoring of key parameters in the cladding process (such as, flow rate, powder concentration, etc.) and real-time adjustment of process parameters combined with feedback mechanism can achieve dynamic optimization and control of the quality of the cladding layer. This is to meet the needs of efficient and accurate powder feeding in different application scenarios.

(3) Application of artificial intelligence and deep learning in powder field dynamics prediction: Explore the potential application of artificial intelligence and deep learning techniques in the prediction of complex dynamics behavior of powder fields. Through the training of deep learning models, we learn and understand the nonlinear, high-dimensional dynamic characteristics of the powder flow process and realize the fast and accurate prediction of the velocity field and other key physical quantities. This can not only effectively shorten the simulation time but also provide richer and more accurate data support for process optimization.

## Figures and Tables

**Table 1:** Comparison of the properties of the hard-ball model and the soft-ball model.

Particle Model	Model Parameter	Finesse	Computational Volume
Hard-ball model	Coefficient of elastic recovery	Thick	Few
Soft-ball model	Damping factor, stiffness factor	Finely particulate	Comparatively large
